# Supplementary material for: Predictive patterning via solid-state dewetting of transferred single-crystal films
Source: Nat Commun. 2026 Mar 28;17:4542. doi: 10.1038/s41467-026-70836-y (PMC13194774; doi:10.1038/s41467-026-70836-y)
Supplement: Supplementary file 1 — Supplementary Information [file 41467_2026_70836_MOESM1_ESM.pdf]

<Supplementary Information>

Predictive Patterning via Solid-state Dewetting of Transferred Single-crystal Films

Seungjin Ju[1]†, Sangsun Lee[1]†, Donghwan Kim[1], Hyunsik Kim[1], Jaeho Lee[1,2], Jeong-Hwan Lee[1,2], Wi Hyoung Lee[3], Olivier Pierre-Louis[4], and Jongpil Ye*[1]

[1]Department of Materials Science and Engineering, Inha University, Incheon 22212, Korea

[2]Program in Semiconductor convergence, Inha University, Incheon 22212, Korea

[3]Department of Materials Science and Engineering, Konkuk University, Seoul 05029, Korea

[4]Institut Lumière Matière, UMR5306 Université Lyon 1—CNRS, Villeurbanne 69622, France

† These authors equally contributed to this work.
*Corresponding Author: jpyecs@gmail.com, jpye@inha.ac.kr

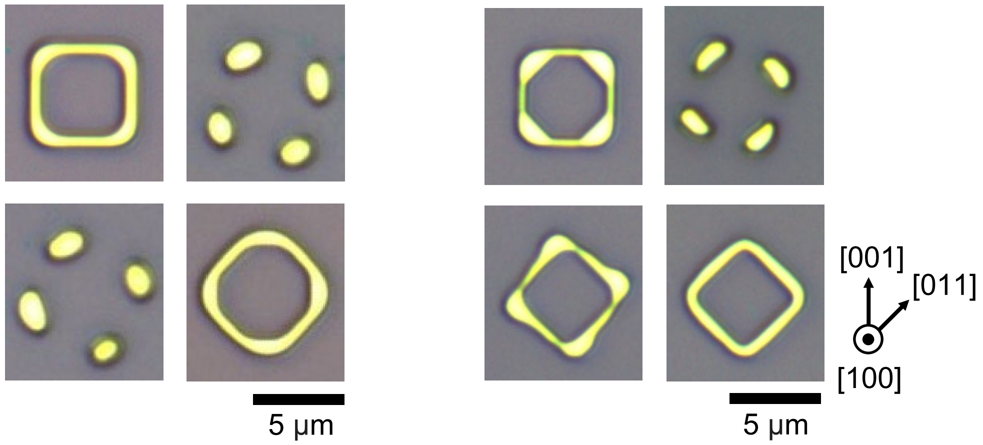

Fig. S1. Enlarged images of dewetting patterns shown in Figs. 2(a) and 2(b).

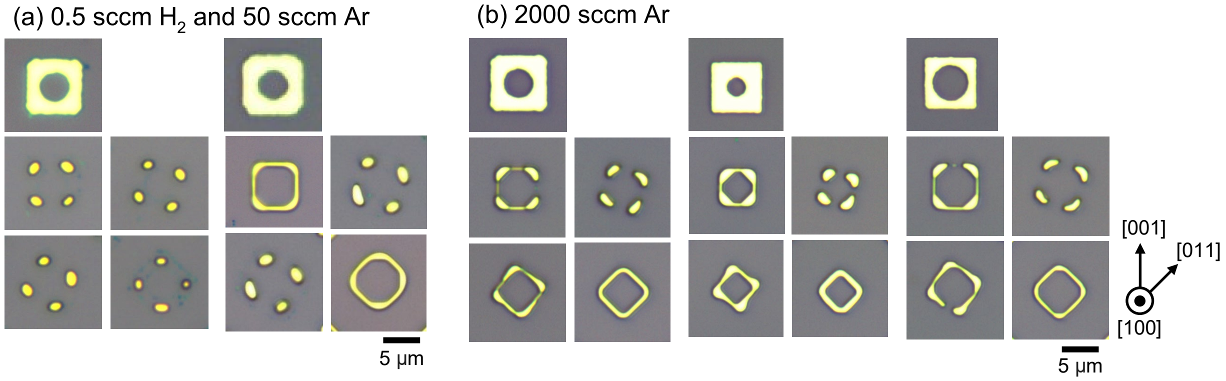

Fig. S2. OM images of various dewetting ring patterns formed in (a) 0.5 sccm H_2 and 50 sccm Ar and (b) 2000 sccm Ar.

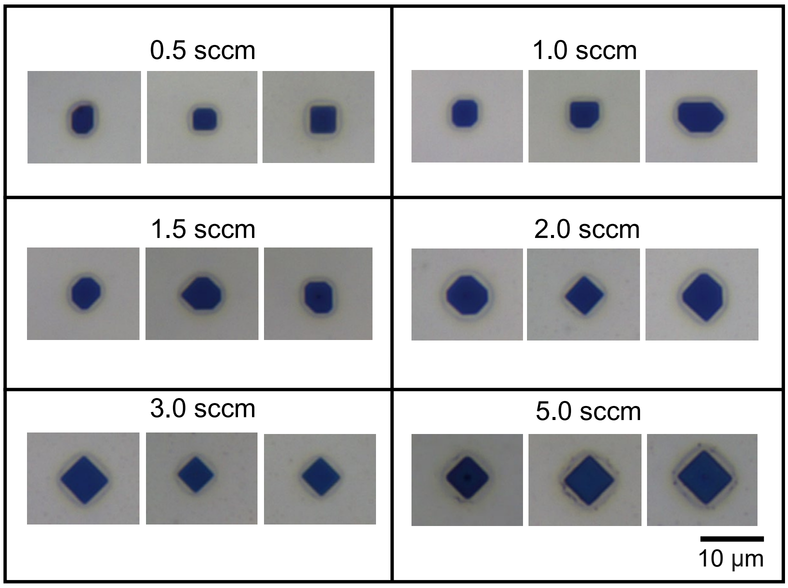

Fig. S3. OM images showing the effects of the hydrogen flow rate on the shape of holes. An Ar flow of 50 sccm was maintained in all cases.

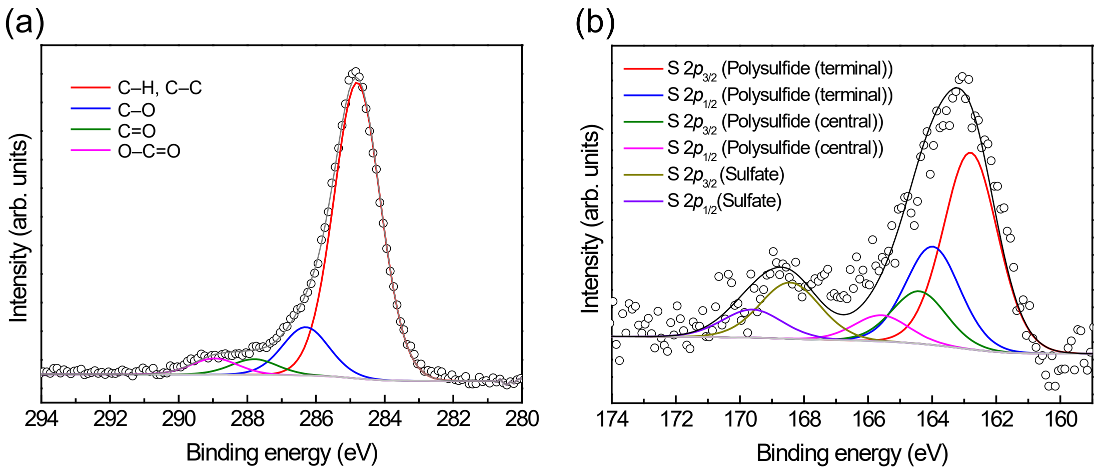

Fig. S4. Representative (a) C 1*s* and (b) S 2*p* XPS spectra measured from annealed Pd(100) films. Source data are provided as a Source Data file.

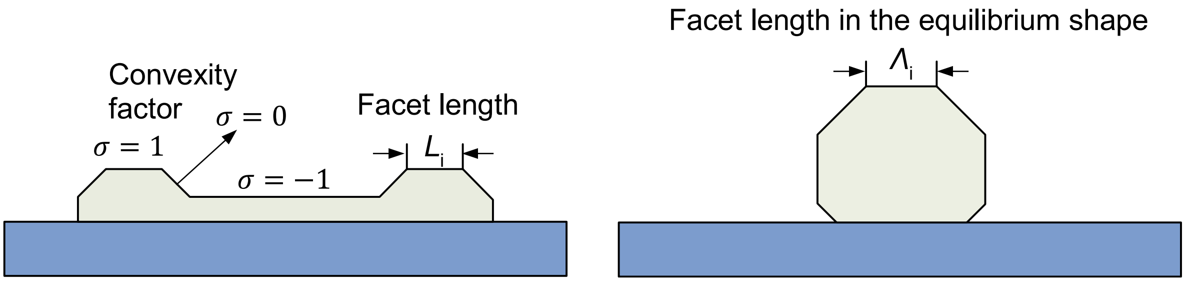

Fig. S5. Parameters determining the weighted curvature of a facet.

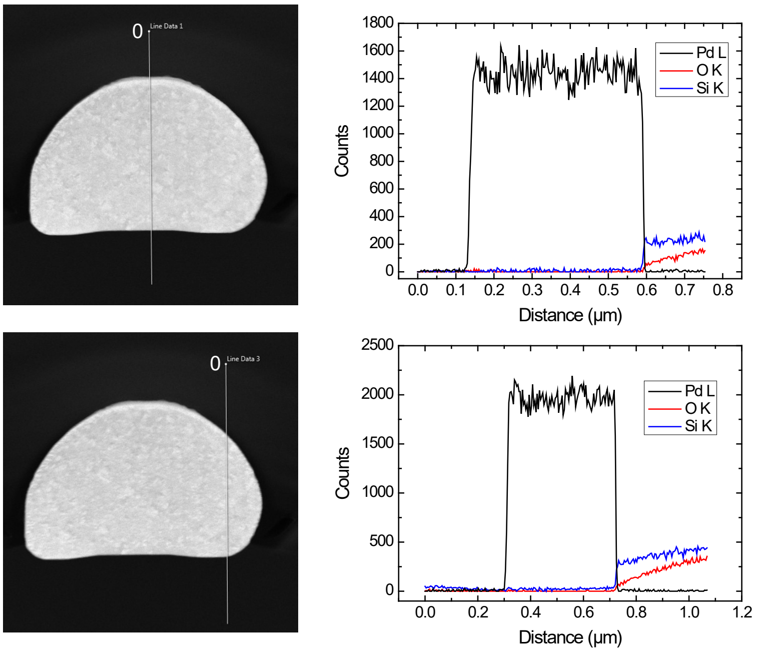

Fig. S6. EDS line-scan results measured across the pattern shown in Fig. 3(d). The measurements were performed from the point denoted as '0' in the TEM images. The origin in the line-scan plots corresponds to this point. Source data are provided as a Source Data file.

Note S1. Extra notes for MD simulation for setting the values of KMC simulation parameters

As mentioned in the main text, MD simulations were conducted to set the values of J, E_S, and ζ, which determine the atomic hopping rate in KMC simulations. The equilibrium lattice constant, determined from NPT simulations, was used to construct all slab structures. The values of J and ζ for Pd were calculated to be 0.274 eV and 0.266, respectively, using the following equations:

$$J = \frac{(E_{1811\_1} - E_{1811\_0})}{2} \quad \text{(S1)}$$

$$\zeta = \frac{(E_{1811\_2} - E_{1811\_0})}{4J} - 1 \quad \text{(S2)}$$

where E_{1811_0}, E_{1811_1}, and E_{1811_2} represent the energies of Pd(1811) slabs and Pd(1811) slabs with two kink pairs of one- and two-atom heights, respectively. The slabs used in the

calculations are shown in Fig. S7. The values of J and ζ were used to calculate the roughening and KMC simulation temperatures as mentioned in the Methods section of the main text. It should be noted that the value of ζ for Pd was not used in the KMC simulations, because the simulation grid was simple cubic rather than face-centered cubic.

The value of ζ for KMC simulations was set by comparing the surface energy ratios of Pd(111) and Pd(100) planes in the NVT MD simulations and the broken bond model including the NN and NNN interactions. The value of E_s was determined by calculating the total energies of the Pd slab, bulk Pd, SiO_2 slab, and Pd/SiO_2 slab, as shown in Fig. S8. The radial distribution functions (RDFs) of Si–Si, Si–O, and O–O in the SiO_2 slab were consistent with those previously reported for amorphous SiO_2, as shown in Fig. S9[1].

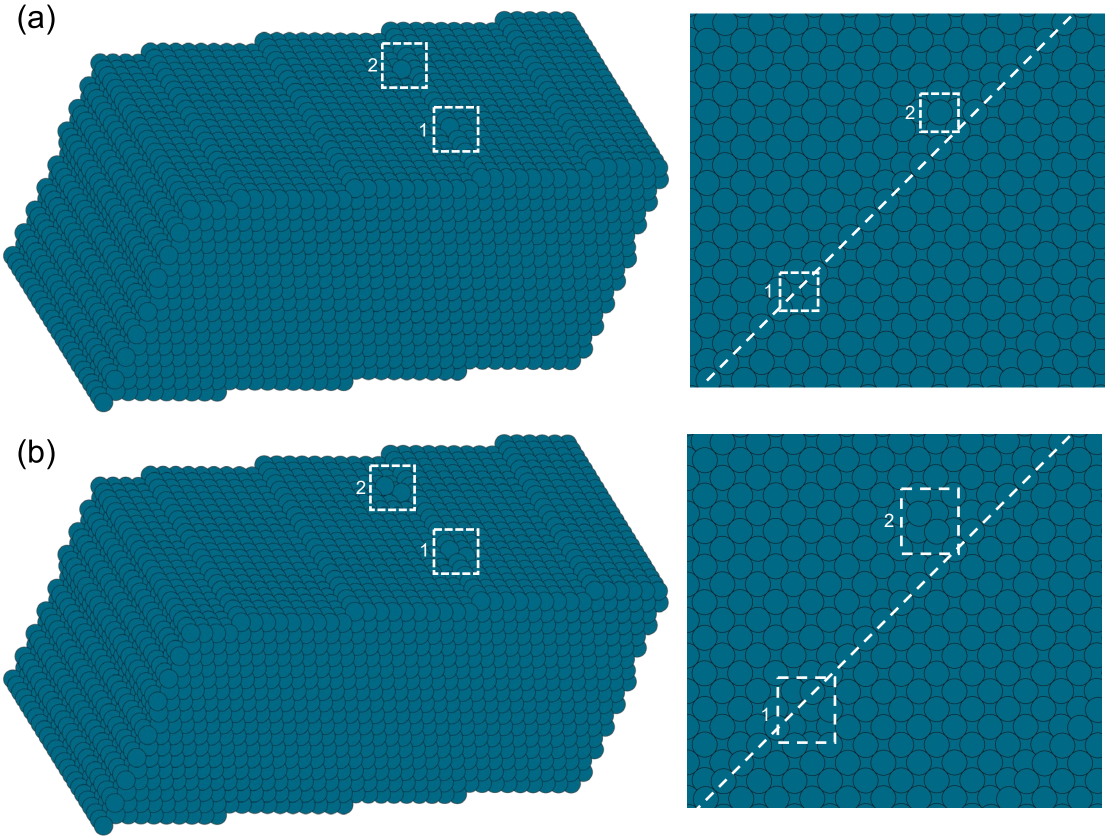

Fig. S7. Pd(1811) Slabs used in the calculations of kink formation energies. (a) Slab with two kink pairs of one-atom height. (b) Slab with two kink pairs of two-atom height. The kink pairs were formed by moving (a) one or (b) two atoms from site 1 to site 2 indicated in the figures. A magnified top view is shown on the right of each figure. The white dashed lines in the top-view images indicate where the atomic step is located.

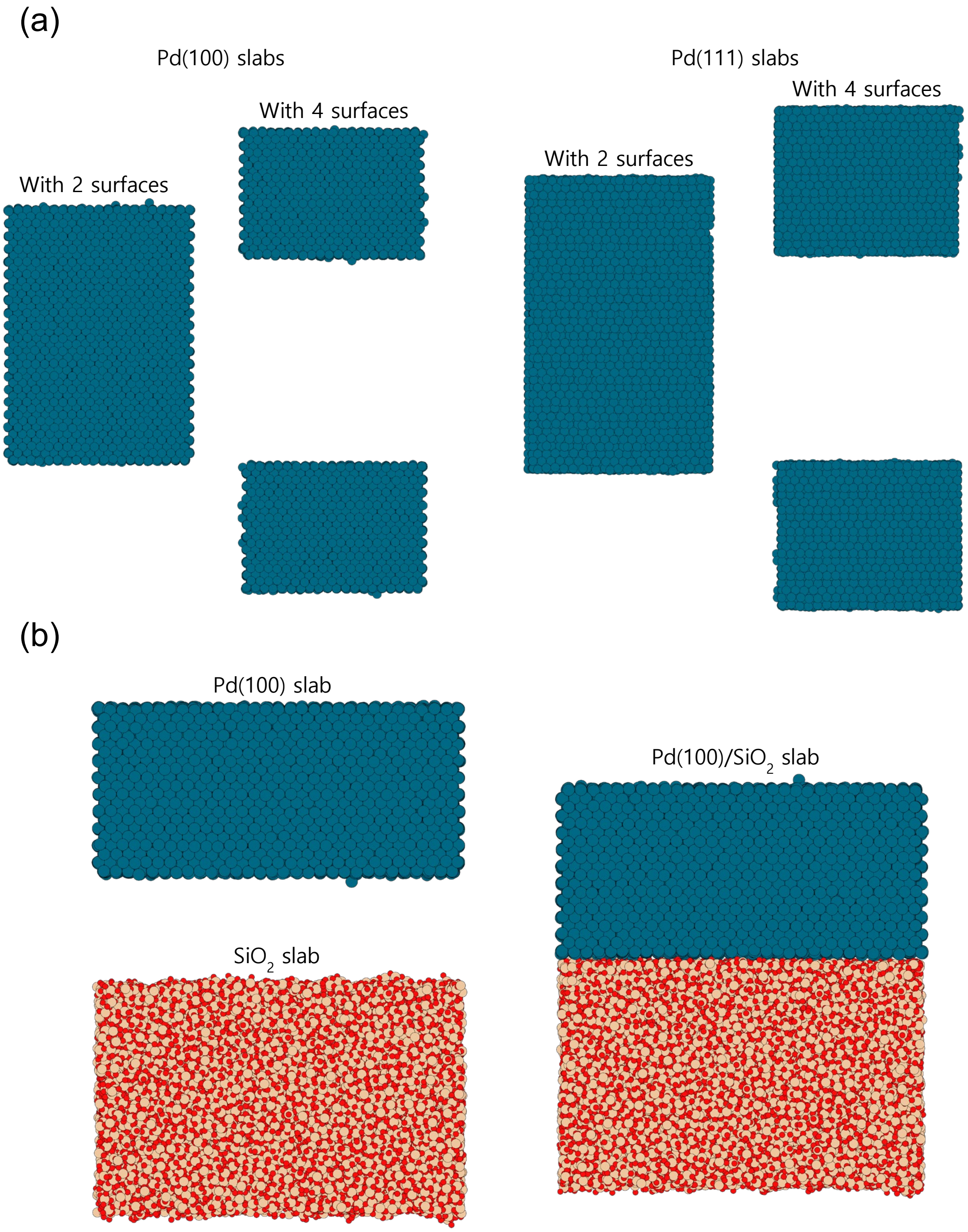

Fig. S8. Slabs used in the NVT MD simulations for setting the values of (a) ζ and (b) E_S^* in KMC simulations.

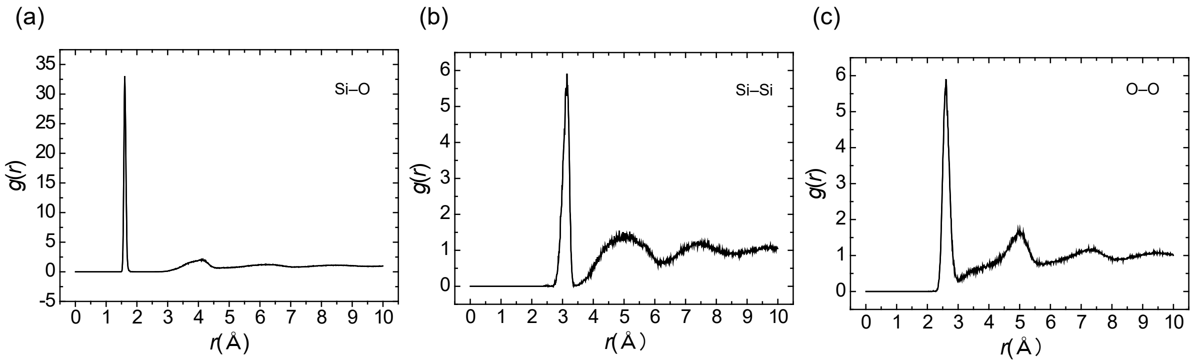

Fig. S9. RDFs of (a) Si–O, (b) Si–Si, and (c) O–O in amorphous SiO_2 slabs created via NVT MD simulations. Source data are provided as a Source Data file.

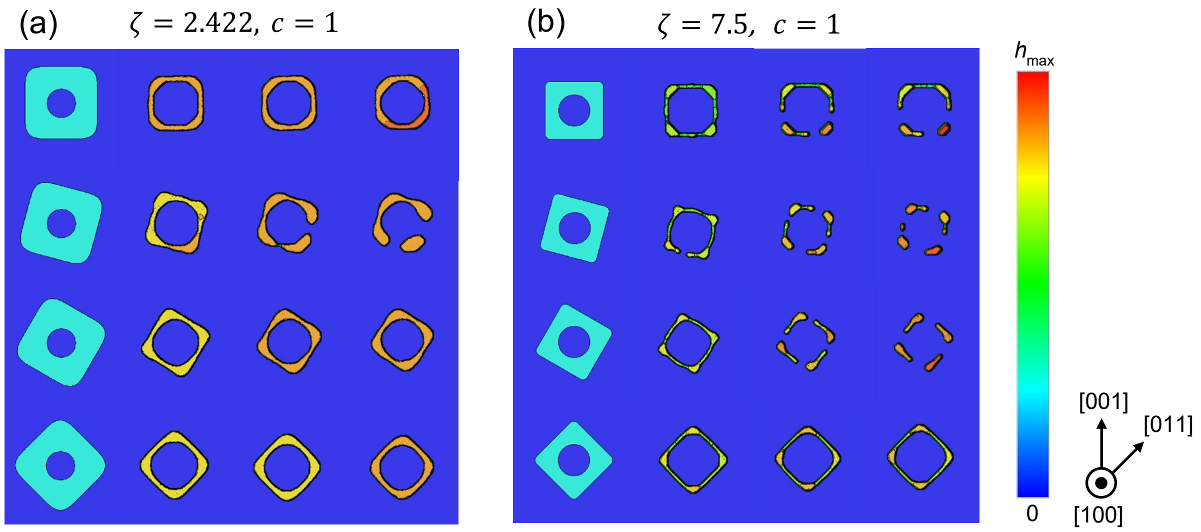

Fig. S10. KMC simulation results obtained without applying the fitting factor. The simulation times were set to be nearly the same in the two panels. The color bar represents the height in the unit of simulation grid, with varying maximum values (h_{max}) across different columns. For the first column in both panels, the maximum height is 11. For columns 2–4 in (a), the maximum height is uniformly 13 across all rows. For columns 2–4 in (b), the maximum heights are 18, 17, 16, and 16 for rows 1–4, respectively. The in-plane crystallographic orientations are indicated in the bottom-right corner.

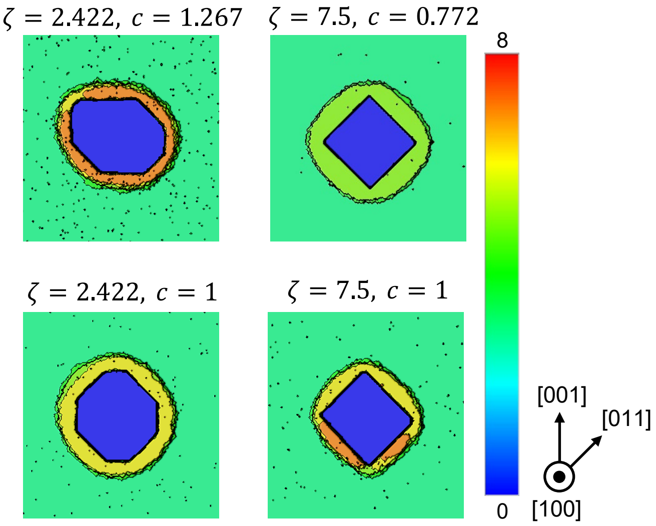

Fig. S11. KMC simulation results for the growth of holes whose initial shapes are circles with a radius of three-grid spacing. The color bar represents the height. The values of ζ and c are shown on top of each panel. Simulation times were set to values that result in hole sizes comparable to those observed in experiments. The in-plane crystallographic orientations are indicated in the bottom-right corner.

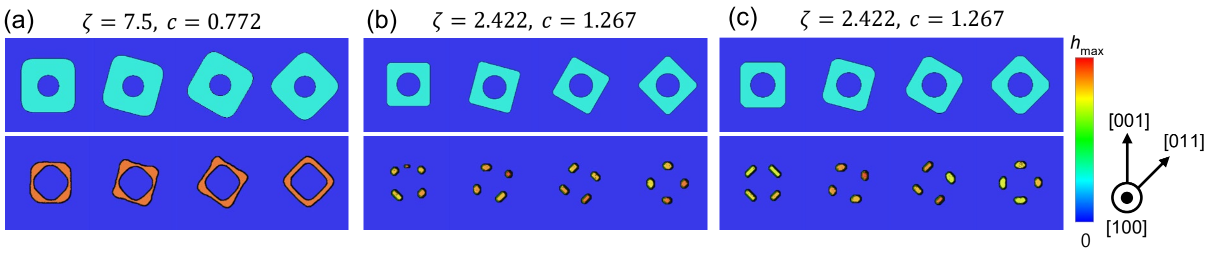

Fig. S12. KMC simulation results for different initial shapes using the parameter sets of Figs. 4(b, c). (a) KMC simulation results for patches whose initial shapes correspond to those in Fig. 4(b). The KMC parameters used here are identical to those in Fig. 4(c). (b) KMC simulation results for patches whose initial shapes correspond to those in Fig. 4(c). The KMC parameters used here are identical to those in Fig. 4(b). (c) KMC simulation results for the patches shown in the left panel of Fig. S2(a), obtained using the same KMC parameters as those in Fig. 4(b). The results were obtained at simulation times nearly the same as those of the rightmost column of Fig. 4(c) for (a), and the rightmost column of Fig. 4(b) for (b) and (c). For all cases, the maximum initial patch height (h_{max}) was 11. The maximum heights of the dewetting patterns were 11 for (a) and 22 for (b) and (c). The in-plane crystallographic orientations are indicated on the right.

Note S2. The effects of stress on the dewetting processes of Pd films.

We calculated the reductions of the surface/interfacial and strain energies during the evolution of a continuous film under stress to a fully stress-relieved particle, as reported by a previous work[2]. In the calculation, it was assumed that the particle is a spherical cap with an equilibrium contact angle of θ.

The reduction of the surface/interfacial energy can be calculated as follows:

$$\begin{aligned} E_{\mathrm{surface}} &= \left(\frac{\pi r^3}{3t}(2+\cos\theta)(1-\cos\theta)^2 - \pi r^2 \sin^2\theta\right)(E_{\mathrm{SV}} - E_{\mathrm{FS}}) \\ &+ \left(2\pi r^2(1-\cos\theta) - \frac{\pi r^3}{3t}(2+\cos\theta)(1-\cos\theta)^2\right)E_{\mathrm{FV}} \\ &= \left[\left(\frac{\pi r^3}{3t}(2+\cos\theta)(1-\cos\theta)^2 - \pi r^2 \sin^2\theta\right)\cos\theta \right. \\ &\left. + \left(2\pi r^2(1-\cos\theta) - \frac{\pi r^3}{3t}(2+\cos\theta)(1-\cos\theta)^2\right)\right]E_{\mathrm{FV}} \end{aligned}$$

$$\begin{aligned} &= \pi E_{\mathrm{FV}} t^2 \left[\left(\frac{\alpha^3}{3}(2+\cos\theta)(1-\cos\theta)^2 - \alpha^2 \sin^2\theta\right) cos\theta \right. \\ &\left. + \left(2\alpha^2(1-\cos\theta) - \frac{\alpha^3}{3}(2+\cos\theta)(1-\cos\theta)^2\right)\right] \end{aligned} \quad \text{(S3)}$$

where E_{FV}, E_{SV} , and E_{FS} represent the energies of film surface, substrate surface, and film-substrate interface, t is the initial film thickness, r is the radius of the spherical cap, and α is the radius-to-thickness ratio.

The reduction of the elastic strain energy during the dewetting can be calculated as follows:

$$\begin{aligned} \Delta E_{\mathrm{strain}} &= -\frac{1-\nu_{\mathrm{f}}}{E_{\mathrm{f}}}\sigma^2 \times \frac{1}{3}\pi r^3 (2+\cos\theta)(1-\cos\theta)^2 \\ &= -\frac{\pi t^3 (1-\nu_{\mathrm{f}})\sigma^2}{3E_{\mathrm{f}}}\left[(2+\cos\theta)(1-\cos\theta)^2 \alpha^3\right] \end{aligned} \quad \text{(S4)}$$

where ν_{f} and E_{f} represent the Poisson's ratio and Young's modulus of the film and σ is the stress exerted on the film.

Hence, the ratio between the two reductions is given by,

$$\frac{\Delta E_{\mathrm{surface}}}{\Delta E_{\mathrm{strain}}} = \frac{\pi E_{\mathrm{FV}} t^2 [A(\theta,\alpha)\alpha^2 + B(\theta,\alpha)\alpha^2]}{-\frac{\pi t^3(1-\nu_{\mathrm{f}})\sigma^2}{3E_{\mathrm{f}}} C(\theta,\alpha)\alpha^2} = -\frac{3E_{\mathrm{f}} E_{\mathrm{FV}}}{t(1-\nu_{\mathrm{f}})\sigma^2} \frac{[A(\theta,\alpha) + B(\theta,\alpha)]}{C(\theta,\alpha)} \quad \text{(S5)}$$

where $A(\theta,\alpha) = \left(\frac{\alpha}{3}(2+\cos\theta)(1-\cos\theta)^2 - \sin^2\theta\right)\cos\theta$,

$B(\theta,\alpha) = 2(1-\cos\theta) - \frac{\alpha}{3}(2+\cos\theta)(1-\cos\theta)^2$, and

$C(\theta, \alpha) = (2 + \cos\theta)(1 - \cos\theta)^2\alpha.$

The value of E_{FV} was obtained from the MD simulation results as mentioned in the main text. The value of $\cos\theta$ can be calculated as follows:

$$\cos\theta \approx \frac{E_{\mathrm{SV}} - E_{\mathrm{FS}}}{E_{\mathrm{FV}}} = \frac{E_{\mathrm{A}}}{E_{\mathrm{FV}}} - 1 \approx -0.698, \quad \text{(S6)}$$

where E_{A} is the adhesion energy.

The values of α have been reported to be approximately 20 and 4, for SOI films and Ag films, respectively. In our calculation, it was set to be 4 considering that it is a value reported for a transition metal film. The Poisson's ratio and Young's modulus of Pd were set to be 0.39 and 120 GPa, respectively. The compressive thermal stress at T is calculated as follows:

$$\sigma_{\mathrm{th}} = \frac{E_{\mathrm{f}}}{1 - \nu_{\mathrm{f}}}(\alpha_{\mathrm{s}} - \alpha_{\mathrm{f}})\Delta T = \frac{1.2 \times 10^{11}}{1 - 0.39}(0.55 - 11.8) \times 10^{-6} \times (T - 298.15) \quad \text{(S7)}$$

where α_{s} and α_{f} are the thermal expansion coefficients of SiO_2 and Pd, respectively. The thermal stress is approximately 1.72 GPa at 800°C, which is significantly greater than the reported values of the yield stress of Pd. Hence, the stress should be plastically relaxed during the heating and approach the yield stress. The yield stresses at room temperature have been reported to be 500 MPa, 250 MPa, and 40 MPa for 90 nm-thick nanocrystalline film, cold-worked bulk, and annealed bulk, respectively.[3,4] The values of $\frac{\Delta E_{\mathrm{surface}}}{\Delta E_{\mathrm{strain}}}$ were calculated to be 12.55, 50.21, and 1961.47, respectively, by entering the three yield stress values mentioned above into Eq. S3-3. Considering that the yield stress decreases with the temperature and grain size, the value in our case should be at least significantly greater than the value for nanocrystalline films. This shows that the dewetting processes in our experiments were primarily driven by the reduction of surface/interfacial energies.

In the statistical mechanics model, the roughening temperature of a film under stress is[5]:

$$T_{\mathrm{R}}(\sigma) = T_{\mathrm{R}}\left[1 - \frac{2a(1 - \nu_{\mathrm{f}}^2)}{E_{\mathrm{f}}}\frac{\sigma^2}{\tilde{\gamma}}\ln\frac{L}{a}\right] \quad \text{(S8)}$$

where T_{R} is the roughening temperature of an unstressed film, a is the lattice constant, L is the length of the film, and $\tilde{\gamma}$ is the surface stiffness. As indicated in the equation, the model predicts that stress lowers the roughening temperature. The values of stress and surface stiffness during the dewetting processes are uncertain at this point. Hence, the quantitative estimation of the lowered roughening temperature was not performed.

Note S3. DFT calculation results for O and H adsorption and its effects on the surface energies of different Pd planes

The most stable adsorption configurations for different Pd planes are shown in Fig. S13. For Pd(311) and Pd(210), a side view is also shown to clarify the configurations. The most stable adsorption configurations were considered in surface energy estimations. As shown in Fig. 4(e), the BEs of O* and H* were consistent in terms of their dependence on the surface orientation of Pd; however, their absolute magnitudes and dependences on the Pd orientations were greater in the case of O*.

As mentioned in the main text, the BEs of O* were used to estimate the surface energies of Pd with O adsorbates. For example, the surface energies of (100) and (111) planes shown in Fig. S13 were estimated by adding the product of the BE and the number of adsorbates per unit area to 0.126 and 0.120 eV/Å^2, which are the surface energies of clean Pd(100) and Pd(111) obtained using MD, as follows:

$$E_{\mathrm{surf+O(111)}} = 0.120 - 1.246 \times \frac{1}{26.929} \times \frac{\theta_{\mathrm{O^*,111}}}{0.25} \quad \text{(S9)}$$

$$E_{\mathrm{surf+O(100)}} = 0.126 - 1.159 \times \frac{1}{31.094} \times \frac{\theta_{\mathrm{O^*,100}}}{0.25} \quad \text{(S10)}$$

where $\theta_{\mathrm{O^*,111}}$ and $\theta_{\mathrm{O^*,100}}$ denote the O coverages on Pd(111) and Pd(100). The coverage was divided by 0.25 because one adsorbate on a 2 × 2 supercell corresponds to 0.25 ML.

The surface energy ratio that corresponds to $\zeta = 7.5$ is 1.119, as mentioned in the main text. Assuming that the two surface coverages are the same, the estimated surface energy ratio becomes 1.119 when the O coverage is 0.143 ML. As mentioned in the Methods section, the BEs can be used in calculating the surface coverages based on Langmuir adsorption theory. According to the calculation, the surface energy ratio becomes 1.119 when the O* coverages on (111) and (100) surfaces are 0.0561 and 0.0227 ML, respectively, at an oxygen partial pressure of ~1.10×10^{-3} atm. The O surface coverage as a function of oxygen partial pressure is shown in Fig. S14(a). There were nearly no changes in the BEs of a single O* when the supercell size was increased to 2 × 4 × 4, which corresponds to 0.125 ML. This indicates that interactions between adsorbates are negligible in the calculation of BEs. Hence, we used the BEs shown in Fig. 4(e) in the analyses of the effects of O adsorption on the surface energies.

H surface coverage as a function of hydrogen partial pressure is shown in Fig. S14(b). Dissociative chemisorption was also assumed in this calculation. As shown in the figure, the surface coverage is calculated to be significantly lower than that of O* at a given partial

pressure due to the lower BEs. Assuming that the ambient in the tube consists of only argon and hydrogen, the hydrogen partial pressures were 0.0566 and 0.0909 atm when the hydrogen flow rates were 3 and 5 sccm in 50 sccm argon, respectively. The corresponding values of ζ were calculated to be ~2.668 and ~2.798, respectively. These increases in ζ are significantly smaller than those by oxygen adsorption, which is qualitatively consistent with the results shown in Fig. S15. Nevertheless, the increases are not sufficiently large to cause the change of hole shapes shown in Figs. 2 and S3. H adsorption was also calculated to decrease the vacancy formation energy, as shown in Fig. S20. This can also enhance the exchange self-diffusion in the <001> directions, making the relative lengths of <001> edges smaller, but its effect is also likely to be limited.

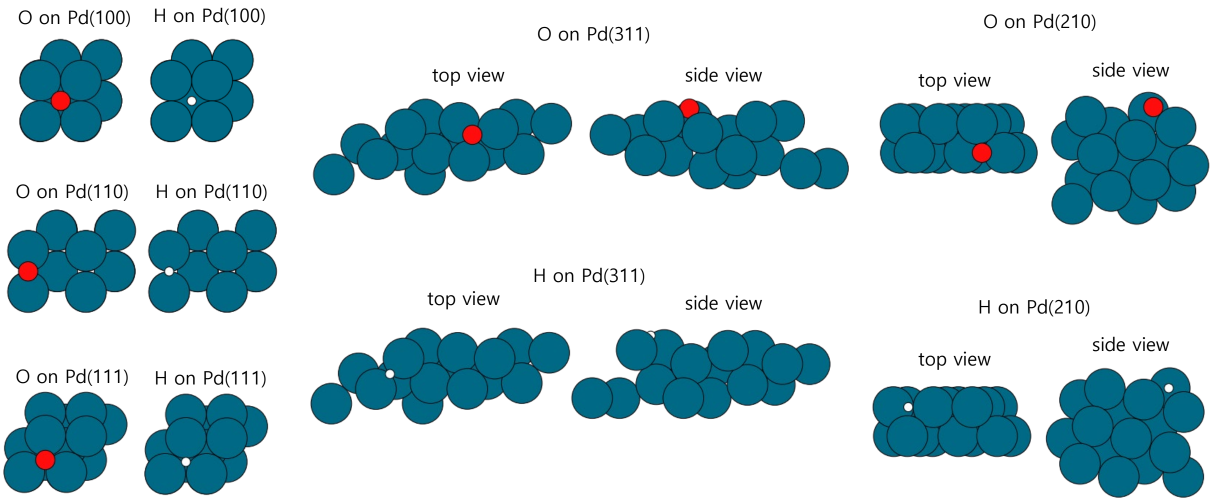

Fig. S13. The most stable adsorption configurations of O* and H* on various Pd slabs.

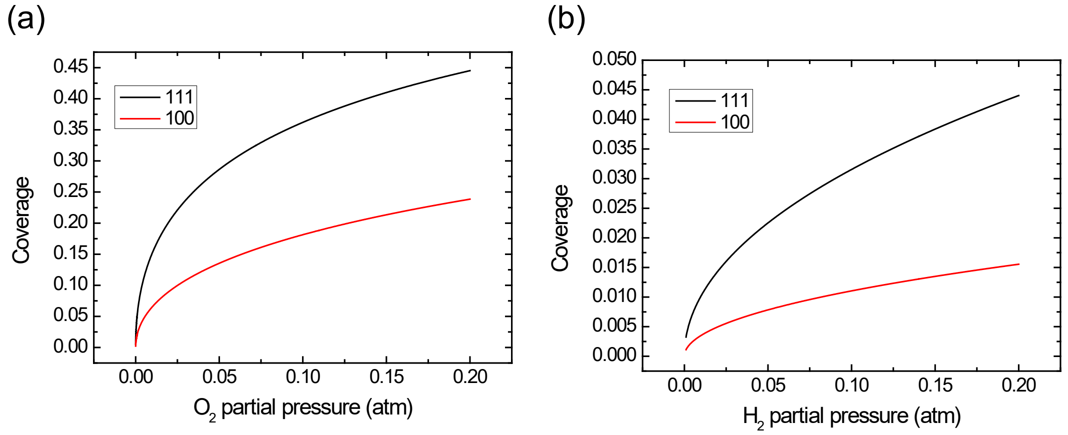

Fig. S14. (a) O and (b) H coverages on Pd(111) and Pd(100) surfaces as a function of O_2 and H_2 partial pressures. Source data are provided as a Source Data file.

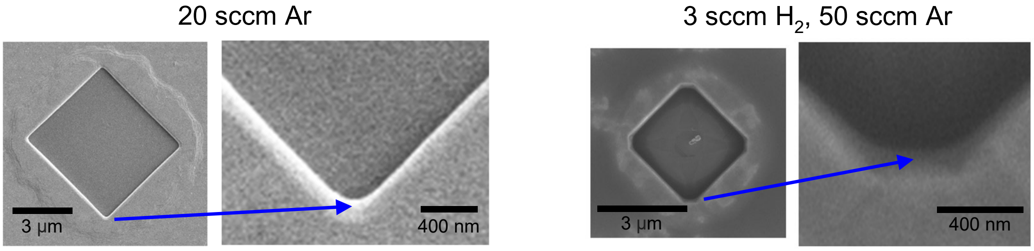

Fig. S15. Enlarged images of holes shown in the insets of Figs. 3(f) and 3(h).

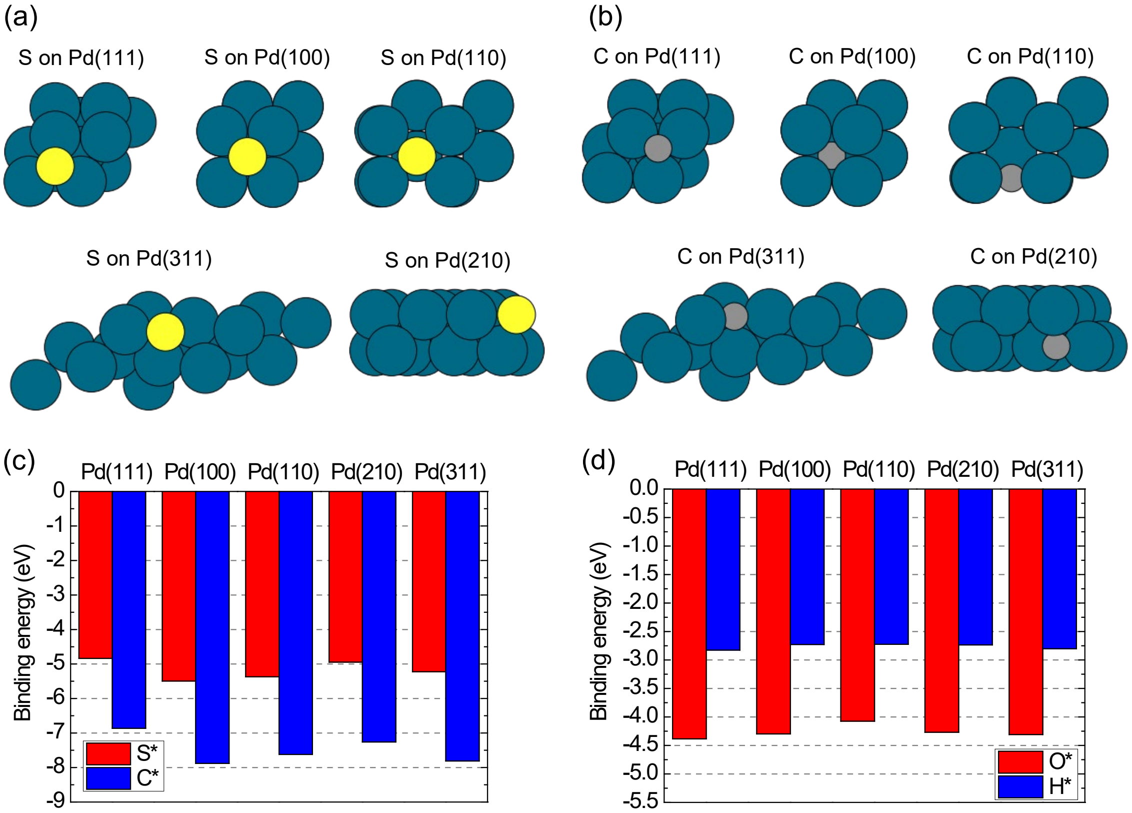

Fig. S16. Adsorption configurations of S* and C* and binding energies of S*, C*, O*, and H* on Pd surfaces. (a, b) The most stable adsorption configurations of S* and C* on various Pd slabs. (c) The BEs of the corresponding S and C adsorbates, calculated with respect to isolated S and C atoms. The BEs were computed as $BE(\mathrm{S}^*) = E(\mathrm{Pd\ slab} + \mathrm{S}^*) - E(\mathrm{Pd\ slab}) - E(\mathrm{S})$ and $BE(\mathrm{C}^*) = E(\mathrm{Pd\ slab} + \mathrm{C}^*) - E(\mathrm{Pd\ slab}) - E(\mathrm{C})$. (d) The BEs of O and H adsorbates with respect to isolated O and H atoms, rescaled from the results shown in Fig. 4(e) for comparison. Source data are provided as a Source Data file.

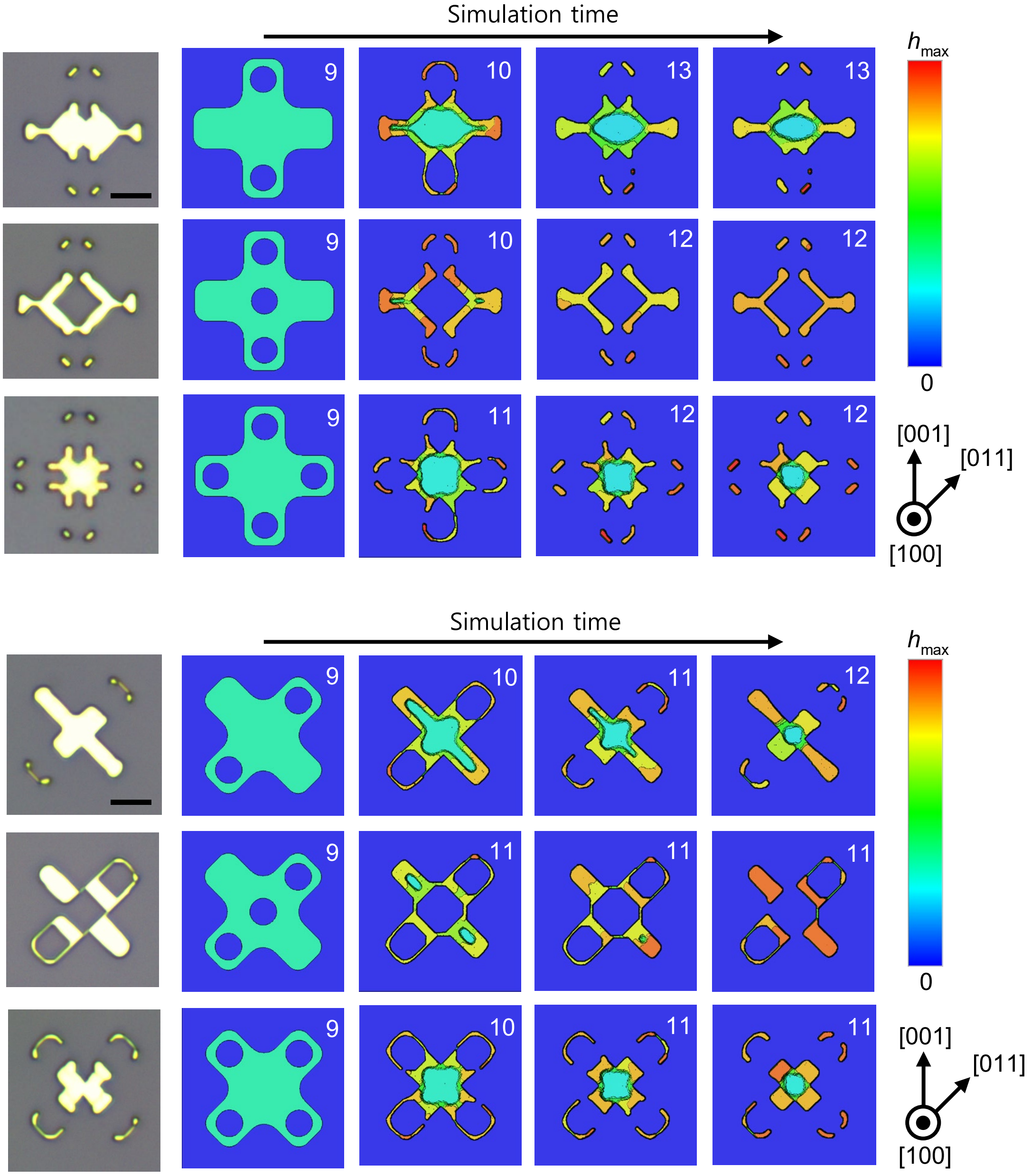

Fig. S17. KMC simulation results for the dewetting of cross patches with internal circular holes. The KMC parameters were set to those shown in Fig. 5. The number in the upper-right corner of each result represents the maximum height (h_{max}) used for the corresponding color bar. Scale bars in the OM images represent 5 μm.

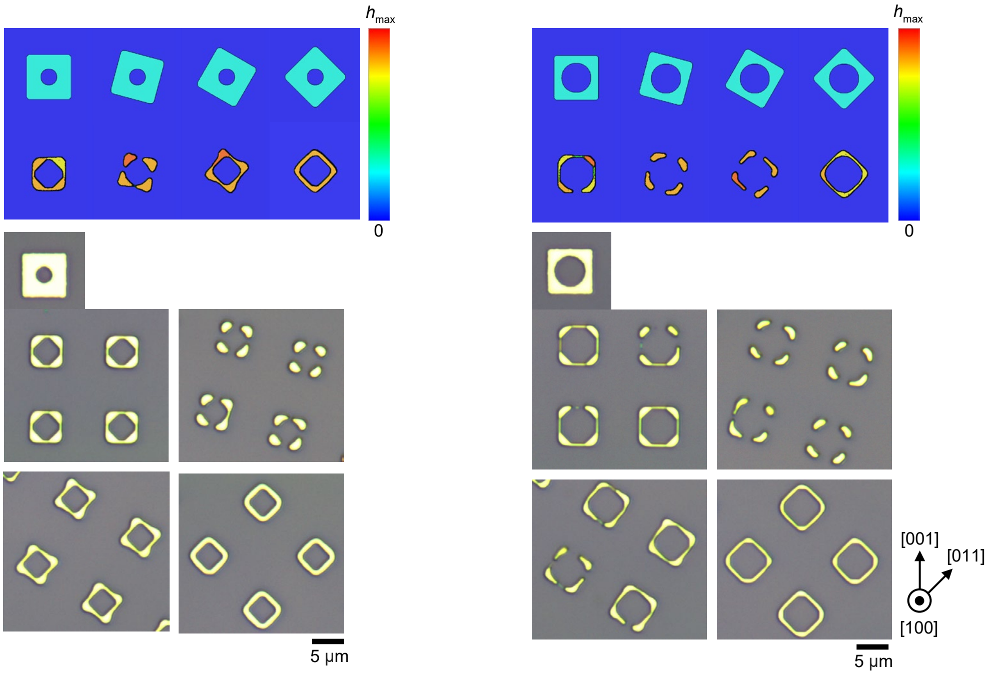

Fig. S18. KMC simulation and experimental dewetting results for the patches in Fig. 6(b) that were not included in Figs. 2(b) and 4(c). The simulation times are nearly the same as those of the rightmost column of Fig. 4(c). For both KMC simulation results, the maximum heights (h_{max}) are 11 for the initial patches and 12 for the dewetting patterns, respectively. The in-plane crystallographic orientations are indicated in the bottom-right corner.

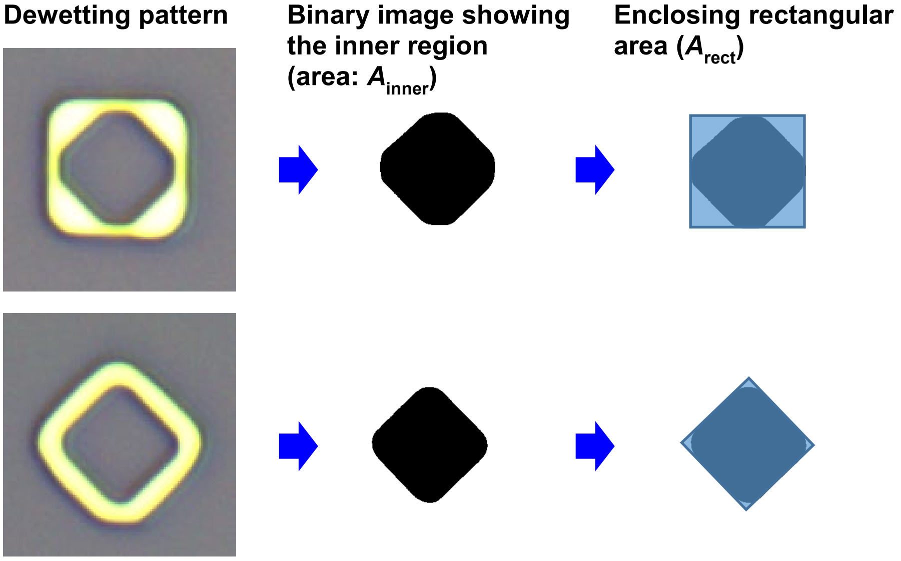

Fig. S19. Procedures for measuring the rectangularities of the inner areas of the ring patterns. The rectangularities were calculated by dividing A_{inner} by A_{rect}. The value of A_{inner} was obtained by counting the number of black pixels in the binary image.

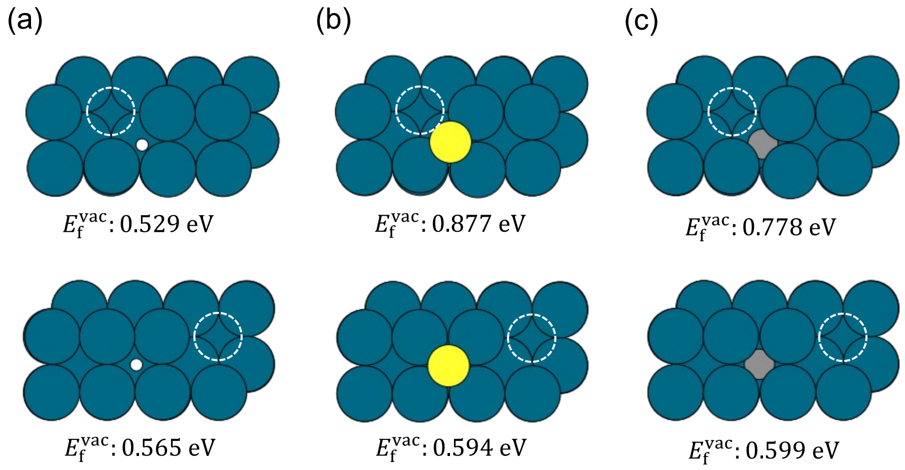

Fig. S20. DFT calculation results showing the vacancy formation energies for (a) H- , (b) S-, and (c) C-adsorbed Pd(100) slabs. Two distinct vacancy positions were considered for each adsorbate.

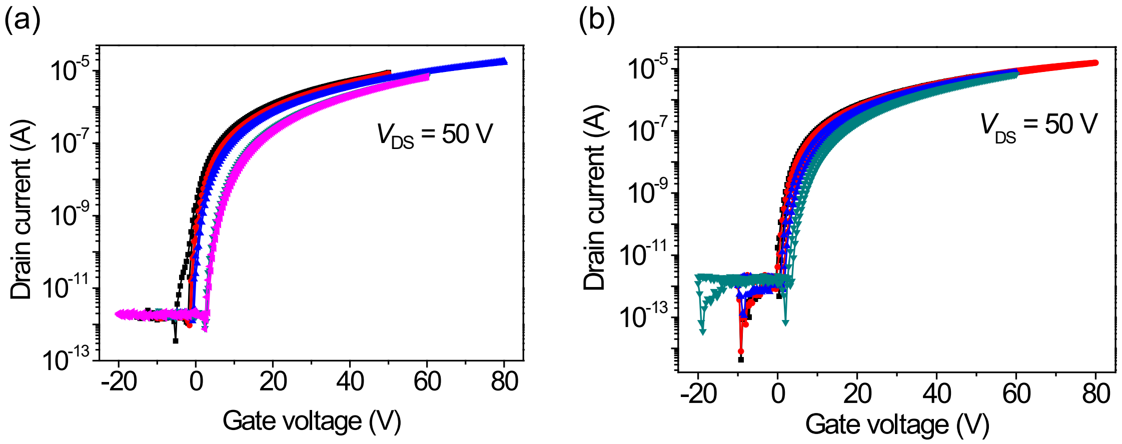

Fig. S21. Transfer characteristics of an IGZO TFT. (a) and (b) show the results of multiple gate-voltage sweeps performed from the ON and OFF states, respectively, measured on the same device used in Fig. 7(e). Source data are provided as a Source Data file.

References

1 Munetoh, S., Motooka, T., Moriguchi, K. & Shintani, A. Interatomic potential for Si-O systems using Tersoff parameterization. *Comp Mater Sci* **39**, 334-339, doi:10.1016/j.commatsci.2006.06.010 (2007).

2 Danielson, D. T., Sparacin, D. K., Michel, J. & Kimerling, L. C. Surface-energy-driven dewetting theory of silicon-on-insulator agglomeration. *J Appl Phys* **100**, doi:Artn 083507
10.1063/1.2357345 (2006).

3 Colla, M. S. *et al.* Dislocation-mediated relaxation in nanograined columnar palladium films revealed by on-chip time-resolved HRTEM testing. *Nat Commun* **6**, doi:ARTN 5922
10.1038/ncomms6922 (2015).

4 Sanders, P. G., Eastman, J. A. & Weertman, J. R. Elastic and tensile behavior of nanocrystalline copper and palladium. *Acta Mater* **45**, 4019-4025, doi:Doi 10.1016/S1359-6454(97)00092-X (1997).

5 Müller, P. & Saúl, A. Elastic effects on surface physics. *Surf Sci Rep* **54**, 157-258, doi:10.1016/j.surfrep.2004.05.001 (2004).
