# Supplementary material for: Predictive patterning via solid-state dewetting of transferred single-crystal films
Source: Nat Commun. 2026 Mar 28;17:4542. doi: 10.1038/s41467-026-70836-y (PMC13194774; doi:10.1038/s41467-026-70836-y)
Supplement: Supplementary file 2 — Description of Additional Supplementary Files [file 41467_2026_70836_MOESM2_ESM.pdf]

## Description of Additional Supplementary Files

**File Name:** Supplementary Software 1

**Description:** Software package for the custom KMC simulations used in this study, including an example executable binary, license file, and README with usage instructions.
