# Supplementary material for: Predictive patterning via solid-state dewetting of transferred single-crystal films
Source: Nat Commun. 2026 Mar 28;17:4542. doi: 10.1038/s41467-026-70836-y (PMC13194774; doi:10.1038/s41467-026-70836-y)
Supplement: Supplementary file 3 — Supplementary Software 1 [file 41467_2026_70836_MOESM3_ESM.zip › software_package/README.pdf]

1. System requirements

The source code for KMC simulation was developed using Fortran. The filename of the source code is dewetnnlitho.for. The executable binary files included in this package were created by editing and compiling the source code using Intel Fortran compiler installed in Ubuntu Linux. The figures included in the manuscript were created by visualizing the simulation output files using OpenDX, which is available for users on Linux systems. Hence, users are advised to run and test the code in Linux systems.

There is no non-standard hardware required to run the code.

2. Installation guide

The source code can be compiled using GNU and Intel Fortran compilers. Installation guides for the compilers can be found in the following links:

GNU: https://fortran-lang.org/learn/os_setup/install_gfortran/

Intel: https://www.intel.com/content/www/us/en/developer/tools/oneapi/fortran-compiler-download.html

GNU gfortran compiler is installed in a few minutes. Installation of Intel compilers takes several tens of minutes.

The authors used OpenDX for visualizing the output files (e.g. fort.20, fort.100, etc.)

OpenDX can be installed very quickly in Ubuntu Linux using the following commands:

```
sudo apt update

sudo apt install dx
```

3. Demo

There are a CSV file and an executable binary file in most subdirectories of examples directories. In the case of binary files for holes, there is no CSV file needed.

A CSV file (bitmap2.csv) contains information on the shape of an initial patch. ‘1’ and ‘2’ in the CSV file represent where the film remains and where the substrate surface is exposed after patterning, respectively.

Users can run the binary code by typing the following command:

```
./dewet > ./dewet.out &
```

By adding ‘&’ at the end of the command, users can run it in background mode.

The binary file loads information on the initial patch from the CSV file and executes KMC simulation. The CSV and binary files should be in the same directory.

There are two types of simulation outputs that are used for analysis and visualization.

If you run the command mentioned above, a file named ‘dewet.out’ is saved. This file contains the simulation step and time.

A group of files named ‘fort.number’ (fort.20, fort.21, etc.) are created after every simulation step. These files contain the information about the height values at each simulation grid.

Users can visualize the height information using OpenDX, which is an open source software package for the visualization of scientific data.

Once users install OpenDX following the aforementioned instruction, it can be executed by typing dx in the terminal.

(1) Press Import Data… button in Data Explorer: the first Data Prompter shows up

(2) Check ‘Grid or Scattered file (General Array Format) in Data Prompter

(3) Select ‘select data file’ in ‘File’ menu of Data Prompter

(4) Press Describe Data… button in the Data Prompter: the second Data Prompter windows shows up

(5) Enter the grid size (500 x 500 in most cases)

(6) Select ‘save as’ in ‘File’ menu of the same Data Prompter

(7) Enter the file name (e.g. image20.general) in the selection text box.

(8) Press ‘Visualize Data…’ button in the first Data Prompter.

(9) Visualized data shows up

Users can check the initial patch by visualizing fort.20, which is the first output file, by following the procedure mentioned above.

The number in an output file name represents the step number. Please note that the step number 20 is the first step.

Users can observe some of dewetting patterns presented in Figs. 4 (rings), 5 (crosses), and S7 (holes) of the manuscript by running the binary files in the examples directory. Please check the pattern evolution by visualizing the output files.

Running times for the demo binaries are expected to range from a few minutes to several tens of minutes on a normal Linux machine.

4. Instructions for use

The source code is included in the source directory. The source file was used to create binary files included in the crosses1-4 and rings1-2 subdirectories of the examples directory.

The source code can be compiled by the following command:

GNU compiler: `gfortran dewetnnlitho.for -o dewet`

Intel compiler: `ifx dewetnnlitho.for -o dewet`

Users can find the lines where the values of ζ, E_s, and T_{KMC} can be changed.

```
parameter(zeta=7.5)

parameter(es=0.850*(1.+4.*zeta),temp=0.068*(1.+4.*zeta),isub=1)
```

If users want to change other simulation conditions, please refer to the comments shown in the top of the source code.

If users want to simulate the dewetting of patches of their own designs, corresponding CSV files should be created. One way to create CSV files is as follows:

(1) Create a bitmap image that defines the top view of a patch. Areas covered by the film are filled with black, while other areas are left white. Set the size of a patch in pixels to make the size-to-thickness ratio the same as the experimental value.

(2) Write a code that reads the bitmap image and create a CSV file in the aforementioned format (a sample executable binary can be provided upon request).
